# Effect of Malathion on Reproductive Parameters of Engorged Female *Rhipicephalus* (*Boophilus*) *microplus* Ticks of Punjab Districts, India

**DOI:** 10.1155/2015/893752

**Published:** 2015-10-15

**Authors:** Jyoti, N. K. Singh, Harkirat Singh, S. S. Rath

**Affiliations:** Department of Veterinary Parasitology, College of Veterinary Science, Guru Angad Dev Veterinary and Animal Sciences University, Ludhiana 141004, India

## Abstract

The present study was aimed at evaluating effects of malathion on the various reproductive parameters, namely, egg mass weight (EMW), reproductive index (RI), percentage inhibition of oviposition (%IO), and hatchability percentage of eggs of *Rhipicephalus* (*Boophilus*) *microplus* (Canestrini 1887) females from 19 districts of Punjab, India. The effect on various parameters was found to be dose dependent and more discernible upon exposure to higher concentrations. Complete cessation of egg laying was recorded in tick isolates on exposure to 5000 ppm and above. The values of %IO ranged in 4.4–68.6, 25.2–76.2, 35.6–100.0, 45.7–100.0, and 71.4–100.0 in groups treated with 1250, 2500, 5000, 10000, and 20000 ppm of malathion, respectively. A low hatching % was recorded in eggs of all treated female ticks in comparison to control treated with distilled water and complete inhibition of hatching was recorded at 10000 ppm and above. However, the survival of the hatched larvae was not affected and was similar to control group. The results of the current study can be of immense help in formulation and implementation of effective tick control measures.

## 1. Introduction

One-host cattle tick,* Rhipicephalus *(*Boophilus*)* microplus *(Canestrini 1887), is an economically important ectoparasite of livestock and creates major problem for milk producers in tropical and subtropical countries including India. It causes severe economic losses by blood loss, reduction in weight gain, and direct damage to skin and hides and also by serving as a vector of various economically important infectious diseases [1]. It is the most prevalent tick infesting all age groups of domestic livestock in various agroclimatic zones of Punjab state, India [2, 3]. The global losses due to ticks and tick borne diseases (TTBDs) were estimated to be between US$ 13.9 and 18.7 billion annually [4] while in India the cost of controlling TTBDs has been estimated to be US$ 498.7 million/annum [5].

The control of this parasite is mostly based on the large scale repeated use of chemical acaricides, namely, organophosphates (OP), synthetic pyrethroids (SP), amidines, and macrocyclic lactones (ML) [6]. Pereira et al. [7] reported that to control* R*. (*B*.)* microplus*, it is necessary to consider that only 5% of parasites are located on the host, so the remaining 95% remain in the environment. Accordingly, several studies [8–10] have emphasized that the successful control of a tick population is related not only to the efficacy of an acaricide but also to the deleterious effects that these active agents cause over tick populations in the field, especially over the reproductive parameters of engorged* R*. (*B.*)* microplus* females.

In India, about 60% of livestock is reared by small and marginal farmers and use of various OP compounds (diazinon and malathion) is very common for the control of livestock and poultry pests [1]. OP compounds are also used against agriculturally important pests and for mass eradication of mosquito larvae in their breeding places [11]. A number of studies have shown development of OP resistance in* R*. (*B.*)* microplus *[12, 13] particularly in Punjab state [14, 15]. However, data on effect of malathion (OP) on the reproductive parameters of ticks indicating its overall tick control efficacy besides causing tick mortality is currently lacking. Based on these observations, the present study aimed to evaluate deleterious effects of malathion on the reproductive parameters of engorged* R*. (*B.*)* microplus* females that had detached from naturally infested cattle.

## 2. Materials and Methods

### 2.1. Location, Geography, and Climate of Study Area

Punjab state is located in the northwest region of India which extends from the latitudes 29.30°N to 32.32°N and longitudes 73.55°E to 76.50°E. It covers a geographical area of 50,362 km^2^ and lies between altitudes 180 and 300 m above sea level. Average rainfall in state is 565.9 mm ranging from 915 mm in north to 102 mm in south with moderately humid climate.

### 2.2. Collection of Ticks

Fully engorged* R*. (*B.*)* microplus* adult female ticks were collected from the dairy sheds of nineteen districts (Amritsar, Barnala, Bathinda, Faridkot, Fatehgarh Sahib, Ferozepur, Gurdaspur, Hoshiarpur, Jalandhar, Kapurthala, Ludhiana, Mansa, Moga, Muktsar, Pathankot, Patiala, Rupnagar, Sangrur, and SBS Nagar) of Punjab, India. The ticks were collected in separate vials, closed with muslin cloth to allow air and moisture exchange, brought to the Entomology Laboratory, Department of Veterinary Parasitology, College of Veterinary Sciences, GADVASU, Ludhiana, and kept at 28 ± 1°C and 85 ± 5% relative humidity (RH).

### 2.3. Acaricide

Technical grade (97.9%) malathion (AccuStandard Inc., USA) was used to prepare the stock solution of 10,000 ppm in methanol. For the bioassay, different concentrations of malathion (1250, 2500, 5000, 10000, and 20000 ppm) were prepared in distilled water from the stock solution and tested against the various field isolates of* R*. (*B.*)* microplus*.

### 2.4. Adult Immersion Test (AIT)

It was conducted as per the method of Drummond et al. [16]. Briefly, 120 engorged females for each isolate were randomly separated into groups of ten (10). A dose-dependent response study was conducted by immersing adult ticks for two minutes in various concentrations of malathion. Two replicates, with 10 engorged females per replicate, were performed for each concentration. Control ticks were immersed in distilled water. After immersion, the ticks were dried on filter paper with the help of paper towels and placed in sterile Petri dishes for complete drying. Afterwards, the ticks were weighed, were transferred to individual glass tubes covered with muslin cloth, and were kept in incubator maintained at 28 ± 1°C and 85 ± 5% RH. The adult ticks which survived the exposure of drug laid eggs which were allowed to hatch to larvae under similar conditions of incubation. The ticks which did not oviposit even after 14 days posttreatment were considered dead and the following parameters were compared:

- Weight of engorged female.
- Egg mass weight (EMW) laid by the live ticks, recorded at 14 days after treatment.
- Reproductive index (RI) = egg mass weight/engorged female weight.
- Percentage inhibition of oviposition (%IO) = [(RI control − RI treated)/RI control × 100].
- Hatching percentage of eggs.

Dose response data of RI and %IO were analyzed by probit method [17] using GraphPad Prism version 4.0, San Diego, CA, USA. The data were statistically analyzed using a one-way analysis of variance (ANOVA) with group multiple comparisons by Tukey's test (GraphPad Prism 4).

## 3. Results and Discussion

The effect of exposure of increasing concentrations of malathion on reproductive parameters of engorged* R*. (*B.*)* microplus* ticks, namely, egg mass weight (EMW), reproductive index (RI), percentage inhibition of oviposition (%IO), and hatching percentage, was studied by AIT. The regression graph of these reproductive parameters of treated ticks was plotted against log values of progressively increasing concentrations of malathion (Table 1). A negative dose-dependent slope was recorded for mean EMW in all tick isolates because with the increasing concentrations of malathion the surviving ticks laid significantly (*p* > 0.05) fewer eggs. Consequently, the mean RI of treated ticks showed a decreasing dose-dependent response and a negative slope was recorded. Results thus indicate that although the increase in concentration of malathion may have not caused one hundred per cent mortality in ticks, the surviving ticks showed a significant decrease (*p* > 0.05) in their efficiency to convert their live weight into egg mass. Also, a dose-dependent significant increase (*p* > 0.05) in the mean %IO of treated ticks along with a positive slope was recorded.

The effect of malathion on the average EMW of treated ticks was recorded to be dose dependent and the decrease was more pronounced at higher concentrations. Complete cessation of egg laying was recorded at 5000 ppm in one isolate, 10000 ppm in four isolates, and 20000 ppm in six isolates, whereas eight isolates laid eggs even upon exposure to the highest concentrations (Table 2). Similarly, details of the effect of malathion on the RI of treated ticks are presented in Table 3. The values of RI ranged from 0.0 to 0.33 in ticks exposed to the recommended concentration of malathion (5000 ppm) used in field conditions. The values of %IO ranged in 4.4–68.6, 25.2–76.2, 35.6–100.0, 45.7–100.0, and 71.4–100.0 in groups treated with 1250, 2500, 5000, 10000, and 20000 ppm of malathion, respectively (Table 4).

The hatching percentage of eggs was determined by visual estimation and a dose-dependent effect was recorded. A low hatching percentage was recorded in eggs laid by all malathion treated female ticks in comparison to control ticks treated with distilled water. Complete inhibition of hatching was recorded in eggs laid by all ticks treated with concentrations of 10000 ppm and above; however, the survival of the hatched larvae was not affected by malathion treatment and was similar to control group.

The absence of studies conducted with the malathion that was used in the present study, regarding its effects on reproductive parameters of fully engorged* R*. (*B.*)* microplus *females, does not allow a comparison with our obtained results. Most trial studies using chemical acaricides are conducted to detect the resistance of* R*. (*B*.)* microplus *to these compounds using* in vitro *methodologies, such as the Adult Immersion Test (AIT), Larval Packet Test (LPT), or the Larval Immersion Test (LIT), recognized by the Food and Agriculture Organization as a standard for the evaluation of efficacy or resistance [18–21].

Further, in the present study, technical grade malathion was selected over commercial formulation for the bioassay because commercial products are prepared with many proprietary ingredients and it is difficult to assess the responses due to active ingredients [22]. For the preparation of stock solution, technical grade malathion was dissolved in 100% methanol and the working concentrations were prepared using water. The use of organic solvents facilitates the adsorption of compound over the surface area of target biological materials and possibly enhances penetration of active ingredients of the acaricide across the exoskeleton [23].

Extensive experience in the field has led to suggestions that the use of strictly managed, uninterrupted, short-interval treatments at recommended concentrations is a reliable means of avoiding or delaying resistance. However, it has also been proposed that intermittent use of high concentration acaricides to kill ticks with resistant alleles may provide a basic means of delaying resistance [24]. Increased concentration has been used successfully in controlling DDT-, OP-, and SP-resistant strains of* R*. (*B.*)* microplus* [25]. This also helped to prolong the life of OP acaricides, but the potential host toxicity and chemical residue problems now need to be reconsidered before an increased concentration could be used for resistance management. However, use of increased concentrations for treatment of animals shed for the elimination of off-the-host stages of the ticks could be beneficial in tick control as they constitute around 95% of total tick population [7]. Several studies [8–10] have emphasized the successful control of a tick population, due to the deleterious effects that active agents cause over tick populations in the field, especially over the reproductive parameters of engorged* R*. (*B.*)* microplus* females. This would further reduce the number of treatments of the animals and would lead to low residual effects in the milk and meat products thus benefitting the end user health.

Based on this finding, the results that we obtained in this present study regarding the reproductive parameters of fully engorged* Rhipicephalus* (*Boophilus*)* microplus *females might be sufficient to reduce the number of chemical treatments administered to cattle.

## Figures and Tables

**Table 1:** Slope, *R* ^2^, and *p* values of reproductive parameters of different isolates of *R.* (*B.*) *microplus* upon AIT with malathion.

Tick isolates	Variables	Slope ± SE	*R* ^2^	*p* value
Amritsar	EMW	−27.74 ± 4.02	0.94	0.0063
	RI	−0.24 ± 0.03	0.93	0.0074
	IO%	40.20 ± 6.19	0.93	0.0074

Barnala	EMW	−27.42 ± 5.38	0.89	0.0146
	RI	−0.17 ± 0.03	0.89	0.0153
	IO%	31.63 ± 6.31	0.89	0.0153

Bathinda	EMW	−33.02 ± 7.38	0.86	0.0209
	RI	−0.3375 ± 0.07	0.86	0.0222
	IO%	68.18 ± 15.63	0.86	0.0223

Faridkot	EMW	−23.91 ± 5.32	0.87	0.0205
	RI	−0.24 ± 0.053	0.87	0.0199
	IO%	40.91 ± 8.99	0.87	0.0199

Fatehgarh Sahib	EMW	−39.07 ± 6.10	0.93	0.0077
	RI	−0.37 ± 0.06	0.92	0.0088
	IO%	80.00 ± 13.10	0.92	0.0088

Ferozepur	EMW	−15.33 ± 2.53	0.92	0.0091
	RI	−0.16 ± 0.028	0.91	0.0110
	IO%	34.81 ± 6.15	0.91	0.0109

Gurdaspur	EMW	−21.90 ± 10.64	0.58	0.1317
	RI	−0.2336 ± 0.07	0.78	0.0453
	IO%	46.26 ± 13.98	0.78	0.0454

Hoshiarpur	EMW	−37.39 ± 6.69	0.91	0.0113
	RI	−0.2951 ± 0.07	0.84	0.0272
	IO%	65.02 ± 14.31	0.87	0.0200

Jalandhar	EMW	−55.50 ± 7.87	0.94	0.0059
	RI	−0.44 ± 0.06	0.93	0.0065
	IO%	73.37 ± 10.79	0.93	0.0065

Kapurthala	EMW	−22.42 ± 3.90	0.91	0.0105
	RI	−0.23 ± 0.03	0.92	0.0091
	IO%	47.92 ± 7.92	0.92	0.0091

Ludhiana	EMW	−14.31 ± 4.88	0.74	0.0109
	RI	−0.12 ± 0.03	0.81	0.0368
	IO%	22.11 ± 6.13	0.81	0.0367

Mansa	EMW	−0.03 ± 0.01	0.79	0.0417
	RI	−0.20 ± 0.06	0.79	0.0410
	IO%	0.34 ± 0.10	0.79	0.0411

Moga	EMW	−29.56 ± 2.09	0.98	0.0008
	RI	−0.26 ± 0.01	0.98	0.0006
	IO%	49.11 ± 3.27	0.98	0.0006

Muktsar	EMW	−26.55 ± 4.21	0.93	0.0081
	RI	−0.28 ± 0.05	0.89	0.0148
	IO%	51.42 ± 10.15	0.89	0.0148

Pathankot	EMW	−27.03 ± 4.728	0.91	0.0106
	RI	−0.2924 ± 0.05	0.91	0.0114
	IO%	52.78 ± 9.45	0.91	0.0114

Patiala	EMW	−40.03 ± 11.86	0.79	0.0432
	RI	−0.37 ± 0.11	0.79	0.0431
	IO%	82.31 ± 24.36	0.79	0.0431

Rupnagar	EMW	−17.59 ± 2.56	0.94	0.0063
	RI	−0.18 ± 0.02	0.96	0.0037
	IO%	37.63 ± 4.55	0.96	0.0037

Sangrur	EMW	−39.36 ± 12.04	0.78	0.0468
	RI	−0.32 ± 0.10	0.77	0.0500
	IO%	0.55 ± 0.17	0.77	0.0500

SBS Nagar	EMW	−18.58 ± 4.35	0.85	0.0236
	RI	−0.21 ± 0.03	0.92	0.0093
	IO%	50.28 ± 8.37	0.92	0.0093

EMW: egg mass weight; RI: reproductive index; %IO: percentage inhibition of oviposition.

**Table 2:** Effect of malathion on egg mass weight of different isolates of *R.* (*B.*) *microplus*.

Isolate	Average egg mass weight (mg)					
	Malathion concentration (ppm)					Control (DW)
	1250	2500	5000	10000	20000	
Amritsar	29.7	24.2	15.0	0.0	0.0	69.9
Barnala	55.9	39.2	37.3	22.3	23.0	84.2
Bathinda	46.8	31.0	28.5	25.0	0.0	52.8
Faridkot	27.2	17.6	3.5	0.0	0.0	51.6
Fatehgarh Sahib	41.0	35.7	17.5	0.0	0.0	55.2
Ferozepur	27.8	27.3	17.0	14.7	11.0	49.6
Gurdaspur	35.0	26.0	26.4	20.0	0.0	50.1
Hoshiarpur	35.0	27.0	28.5	16.3	0.0	50.1
Jalandhar	68.1	53.2	22.0	22.0	0.0	77.9
Kapurthala	26.8	14.0	11.0	0.0	0.0	47.6
Ludhiana	20.8	16.6	15.5	15.0	0.0	76.9
Mansa	65.3	30.8	37.3	18.5	15.0	99.6
Moga	50.8	41.2	33.1	20.8	16.5	65.1
Muktsar	45.5	29.0	23.5	16.0	12.0	56.5
Pathankot	37.4	21.5	17.1	15.0	0.0	56.5
Patiala	43.3	34.1	0.0	0.0	0.0	46.6
Rupnagar	29.3	27.9	17.0	13.5	10.0	50.4
Sangrur	60.9	30.8	13.33	14.0	10.0	72.4
SBS Nagar	29.4	29.0	14.1	11.7	10.0	42.0

**Table 3:** Effect of malathion on RI of field isolates of *R.* (*B.*) *microplus*.

Isolate	Reproductive index (RI)^a^					
	Malathion concentration (ppm)					Control (DW)
	1250	2500	5000	10000	20000	
Amritsar	0.26	0.22	0.14	0.0	0.0	0.61
Barnala	0.34	0.26	0.26	0.14	0.15	0.53
Bathinda	0.47	0.34	0.30	0.27	0.0	0.49
Faridkot	0.28	0.19	0.04	0.0	0.0	0.60
Fatehgarh Sahib	0.39	0.35	0.17	0.0	0.0	0.47
Ferozepur	0.29	0.29	0.18	0.15	0.12	0.46
Gurdaspur	0.34	0.23	0.25	0.21	0.0	0.51
Hoshiarpur	0.39	0.29	0.33	0.19	0.0	0.51
Jalandhar	0.59	0.42	0.17	0.18	0.0	0.60
Kapurthala	0.28	0.15	0.12	0.0	0.0	0.49
Ludhiana	0.18	0.13	0.12	0.11	0.0	0.56
Mansa	0.37	0.20	0.26	0.11	0.10	0.60
Moga	0.46	0.40	0.29	0.20	0.15	0.54
Muktsar	0.47	0.32	0.18	0.15	0.12	0.56
Pathankot	0.41	0.24	0.21	0.17	0.0	0.55
Patiala	0.41	0.32	0.0	0.0	0.0	0.46
Rupnagar	0.32	0.29	0.19	0.14	0.11	0.49
Sangrur	0.53	0.27	0.14	0.15	0.11	0.58
SBS Nagar	0.35	0.31	0.18	0.13	0.12	0.43

^a^Reproductive index (RI) = egg mass weight/engorged female weight.

**Table 4:** Effect of malathion on %IO of different isolates of *R.* (*B.*) *microplus*.

Isolate	Percentage inhibition of oviposition (%IO)^a^					
	Malathion concentration (ppm)					Control (DW)
	1250	2500	5000	10000	20000	
Amritsar	57.2	64.6	76.4	100.0	100.0	0.0
Barnala	36.3	50.8	51.1	73.5	72.6	0.0
Bathinda	4.4	31.3	39.4	45.7	100.0	0.0
Faridkot	54.0	68.7	93.8	100.0	100.0	0.0
Fatehgarh Sahib	16.9	25.2	63.9	100.0	100.0	0.0
Ferozepur	36.6	37.5	61.4	67.4	74.1	0.0
Gurdaspur	32.9	53.7	49.9	58.8	100.0	0.0
Hoshiarpur	22.2	42.8	35.6	82.9	100.0	0.0
Jalandhar	8.9	30.7	71.1	69.8	100.0	0.0
Kapurthala	42.3	68.9	75.0	100.0	100.0	0.0
Ludhiana	68.6	76.2	78.3	79.9	100.0	0.0
Mansa	38.6	66.8	56.9	81.0	84.0	0.0
Moga	15.4	26.2	45.5	62.3	71.4	0.0
Muktsar	16.2	42.7	67.2	72.9	78.5	0.0
Pathankot	26.5	56.7	62.9	68.8	100.0	0.0
Patiala	10.9	30.0	100.0	100.0	100.0	0.0
Rupnagar	35.9	41.6	62.5	70.9	77.9	0.0
Sangrur	9.1	53.1	76.8	74.9	81.6	0.0
SBS Nagar	17.9	27.6	57.8	69.6	72.6	0.0

^a^%IO = [(RI control − RI treated)/RI control × 100].
